# Biological, Psychological, Social, and Legal Aspects of Trans Parenthood Based on a Real Case—A Literature Review

**DOI:** 10.3390/ijerph16060925

**Published:** 2019-03-14

**Authors:** Maria-Elisa de-Castro-Peraza, Jesús Manuel García-Acosta, Naira Delgado-Rodriguez, Maria Inmaculada Sosa-Alvarez, Rosa Llabrés-Solé, Carla Cardona-Llabrés, Nieves Doria Lorenzo-Rocha

**Affiliations:** 1 Faculty of Nursing N. S. Candelaria, University of La Laguna, 38010 Tenerife, Canary Islands, Spain; extmcastrop@ull.edu.es (M.E.d.-C.-P.); isalvar@ull.edu.es (M.I.S.-A.); rosallabres@telefonica.net (R.L-S.); carlallabres@gmail.com (C.C-L.); extnlorenzo@ull.edu.es (N.D.L.-R.); 2 Faculty of Psychology, University of La Laguna, 38071 Tenerife, Canary Islands, Spain; ndelgado@ull.edu.es

**Keywords:** transgender person, pregnancy, parenting, assisted reproduction, health legislation, holistic health

## Abstract

Trans men are people who, based on their genitals, were assigned the status of female at birth. However, their identity and their way of living gender do not correspond to the socially established norms. In this paper, we discuss the different perspectives in relation to transgender people and their desire for parenthood. This review, and the basis of this paper, is inspired by the case of a trans man who desired gestation with his own genetic material. He began the cycle of assisted reproduction when he was a legally recognized woman, but that attempt ended with a miscarriage. From that assisted reproduction cycle, four embryos remained frozen. After the failed experience of gestation, the person completed his transition. Now legally a man, he attempted to gestate using his reproductive organs. This literature review aimed to identify relevant studies describing the relationship between transgender person and biological parenthood. This study comprehensively addresses important aspects one should know when considering a transgender pregnancy. These factors include biological, psychological, social, and legal issues. After reviewing the state-of-the-art information on trans parenthood, the main conclusion is that ‘the desire to have a child is not a male or female desire but a human desire’.

## 1. Introduction

Transgender parenthood opens a debate full of complexities at different levels that have not been sufficiently addressed by the scientific community. However, for people who face the decision to transition to another gender, an integral approach to their situation and to their decision to become parents turn out to be central. We present the case of Dan, a Spanish trans man who wants to have offspring. Dan is currently 38 years old. Ten years ago, as a woman, she went to the fertilization unit of her hospital with her previous partner, another woman, because they wanted to have a child. At that time, Dan underwent ovarian stimulation with subsequent extraction of oocytes and fecundation with donor semen. Four embryos were implanted but were eventually rejected and aborted by Dan’s body. Another four embryos currently remain vitrified.

At age 30, Dan started cross-hormone testosterone treatment for a sex change and chest masculinization surgery. After divorcing his previous partner, Dan joined another woman, age 45, with whom he now wishes to have biological offspring. Dan would like to use his frozen embryos, although he does not renounce other ways of parenthood.

According to Spanish legislation, embryos can be used only by the owner couple, in this case, Dan and his first partner. If this does not happen, they must be destroyed, ceded for research purposes, or donated anonymously [1]. In Dan’s case, his ex-partner does not want to use the embryos herself, but she does not want to give them to Dan either. Currently, he is in the middle of a legal battle for the ownership of the vitrified embryos. Regardless of the judicial opinion, however, Dan wants to proceed with the gestation, either with his own genetic material or that of a donor. Dan wants to have offspring, and he has the physical ability to gestate and carry out the pregnancy to term. It is not the first case referenced in the literature. Other trans men have been able to gestate and breastfeed their own children as a result of the reversibility of certain effects of hormone therapy in the absence of the previous surgery. Until a few years ago, female to male (FtM) therapy involved only a decision regarding the possibility of cryopreservation of ovarian tissue to allow another person, always a woman, to gestate. Currently, there is a strong trend against sex reassignment surgeries that imply changes contrary to the biological possibility of having offspring.

The case of Dan does not leave us indifferent. Trans people have had to fight for many years just to have their individual rights recognized in such basic matters as identification related choosing a name, gaining access to health systems, and nondiscrimination due to gender issues as recognized in the Universal Declaration of Human Rights. In the case of gestation, the very rejection suffered by transgender people and the social impact of the image of a pregnant man supposes not only legal and biological issues for the birth parent but for the development and physical well-being of the fetus as well.

Gestational paternity is a reality today. Even though more and more men are becoming pregnant and having children, the specialized scientific literature describing the experience of trans person gestation remains scarce [2,3,4]. Most of the existing publications offer more controversy than they do knowledge and debate. Moreover, the position of the scientific research has focused on aseptic information and on the biological aspects without analyzing the issue from other multiple perspectives. The demand for the democratization of access to these technological advances in assisted reproduction adds new questions to those already existing. With this article, the authors intend to examine the issue from different perspectives in a way that serves as a starting point to understand the situation. Throughout the document, the term trans is used as an inclusive expression of all trans binary and nonbinary identities.

## 2. Materials and Methods

To identify previous research and future research needs, the literature review method was applied. A limited systematic review of the literature was conducted using four extensive databases: Academic Search Complete (EBSCO), PubMed, PsycInfo, and Web of Science (WoS). The aim was to identify relevant studies on the relationship between a transgender person and biological–gestational parenthood.

The search was performed from June to September 2018. It retrieved published articles using keywords related to transgender people and biological parenthood using assisted reproduction techniques, according to the legal background and considering psychosocial issues.

The search query included (but was not limited to) terms: (transsexualism (MeSH) OR transgender person OR transgenderism OR sexisms), (reproductive techniques, assisted (MeSH) OR fertility preservation OR sterilisation), (pregnancy (MeSH) OR parenting (MeSH) OR parenthood OR prenatal education), (health legislation (MeSH) OR family law), and (holistic health (MeSH) OR integrative medicine). The results from the literature search were complemented by the author’s own collections of relevant documents.

All original research articles published in English or Spanish were considered. No restrictions based on year of publication or methodology were applied. No systematic data extraction or quality evaluation of the included studies was employed.

**Figure 1:**
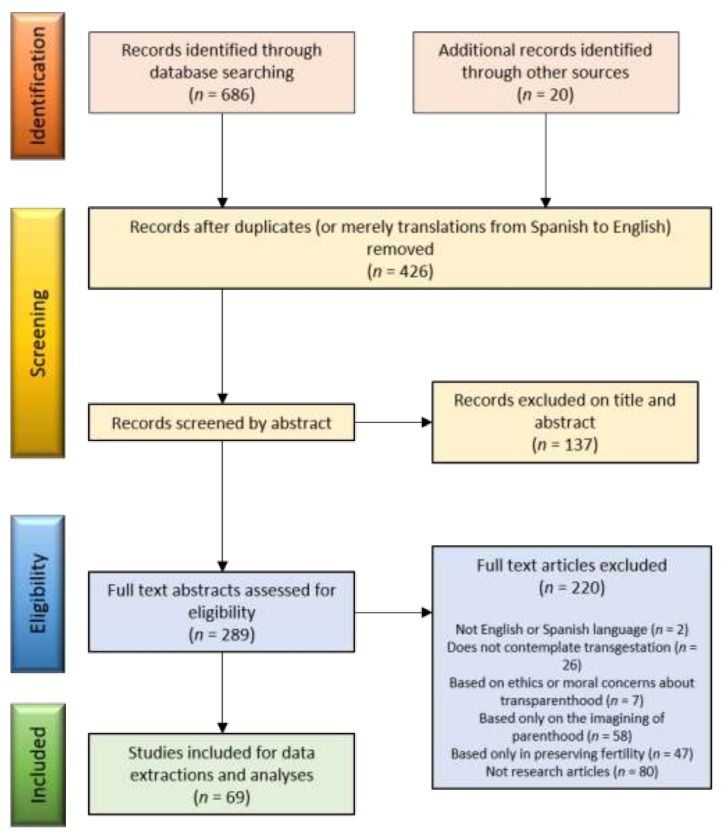
A flowchart showing phases of the literature search for extraction of the most specific literature for the review.

A total of 706 studies with the main objective of transgender parenthood was identified; of these, 69 studies met the inclusion criteria and were used in this literature review. Abstracts were reviewed for relevance, and relevant manuscripts were reviewed in full. Discussion sessions were held to increase the consensus of the group while screening and analyzing. During the consensus meetings, themes were identified through observation and discussion. Refer to Figure 1 to see the flow diagram of the search strategy. In general, the literature search resulted in articles that examined biological (or biomedical) aspects, psychological aspects, social aspects or legal aspects. Particularly this last point, legal aspects, was mainly complex due to the diverse legislations in the different countries. In the most recent years, efforts have been made to more accurately describe the multifaceted interactions between transgender people and biological parenthood. However, authors could not find research that considers holistically all these dimensions.

## 3. Results

An analysis of the data extracted from the 69 included articles revealed four broad themes: Biological or biomedical aspects [3,4,5,6,7,8,9,10,11,12,13,14,15,16,17,18,19,20,21,22,23,24], psychological aspects [3,7,8,9,12,15,16,18,20,25,26,27,28,29,30,31,32,33,34,35,36,37,38,39,40,41], social aspects [2,25,37,42,43,44,45,46,47,48,49,50,51,52,53,54,55,56,57,58] and legal aspects [1,59,60,61,62,63,64,65,66,67,68,69].

**Figure 2:**
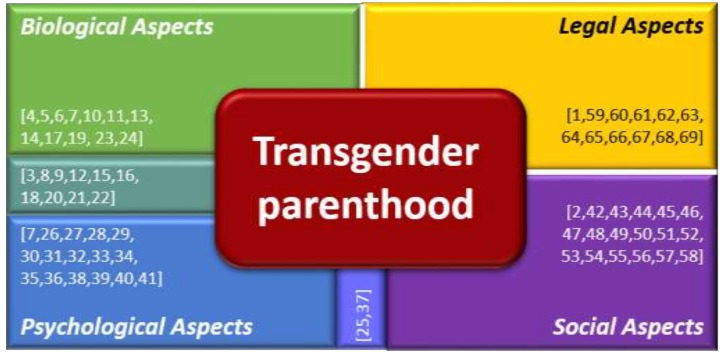
The analysis of the data revealed four broad themes.

Refer to Figure 2 to see and overview of the articles and the theme that they align with.

### 3.1. Biological Aspects

The hormonal and surgical treatments that trans men undergo have devastating effects, including a significant but potentially reversible impact on fertility [3]. Although the cessation of menstruation, which results from the crossed hormonal treatment, occurs within the first 8–12 months [4,5,6], there are studies that show that trans men can have menses and ovulations within the first six months after the interruption of testosterone [7,8]. Regarding reproduction, androgen therapy does not seem to alter the ovarian reserve [9], which shows normal serum levels of the antimüllerian hormone and inhibin-B [10], although this is currently under investigation [4]. Current studies indicate that the best option for the preservation of fertility is the cryopreservation of embryos or oocytes [6,11], but both techniques involve two weeks of controlled ovarian hyperstimulation treatment [9,12]. In addition to cryopreservation, new techniques have been proposed, such as the activation of immature follicles, in vitro maturation of immature oocytes, and even, in a promising future, the manufacturing of artificial gametes from somatic cells, which is important when planning parenthood in men who have undergone radical surgery for gender affirmation [11].

There are several studies that recommend the use of letrozole during the hyperstimulation protocol to avoid high estrogenic levels, decrease the reversibility of the abandonment of testosterone, and help patients to adhere to the treatment [8,10]. The use of letrozol before conception is considered safe given that the evidence does not reveal any relationship with future congenital malformations [13,14].

Testosterone, however, does have teratogenic effects on the fetus. Therefore, trans men should avoid pregnancy during treatment [3,15,16], and testosterone should be stopped when trying to conceive to decrease the risk of teratogenicity [3,7,8].

From a physiological perspective, there are no studies that indicate that a transgender man’s pregnancy differs from that of a cisgender woman. However, there are studies that suggest that long-term testosterone administration in trans men during the reproductive age induces a low proliferative active endometrial rate, fibroids, and hypertrophic myometrium in up to 58% of individuals [5]. A study by Grynberg et al. found that of 112 trans men exposed for at least three years to androgens, only 48% exhibited proliferative endometrium, while the other 44.6% displayed atrophic changes [17]. Because the fertile age of the person does not change, and the possibility of pregnancy depends on the age and the ovarian reserve, as determined by the anti-Müllerian hormone levels, the cumulative pregnancy rate was significantly lower in patients older than 35 years [18]. By contrast, a study conducted by Light et al. revealed that trans men who had never taken hormones were almost three times more likely to become pregnant than were those who had been treated [19]. Despite multiple factors, trans men have successfully conceived and completed a pregnancy after using testosterone [15], and the majority of them became pregnant after four months of trying to conceive [7].

Many of the studies reflect a lack of specialized obstetric care in the case of pregnant transgender men, suggesting that they have little knowledge regarding prepregnancy and perinatal care [3,7,15,20,21]. The use of hormonal treatment with testosterone can influence the way one gives birth. For example, one study revealed that the highest proportion of caesarean births was performed among trans men taking testosterone (36%) versus those who did not (19%) [15]. In addition, the literature indicates that there is a greater proportion of caesareans [7], mainly due to the trans person’s own decision given that the idea of a vaginal delivery with their genitals exposed for long periods of time was perceived as a disturbing experience [22].

Among the cases of transgender pregnancies and well-documented experiences, two are quite notable. On the one hand is the case of Thomas Beatie, a transgender man who became pregnant after suspending his hormonal testosterone treatment that he had been following for two years [23]. In his book “Labour of Love” [24], Thomas Beatie details how he approached and completed his pregnancy, explains that he could have more than one child, and describes his experiences with health professionals, neighbors, and family. On the other hand, we have the case of Trystan Reese, a 34-year-old transgender man who, together with his husband, became pregnant naturally after six months of attempting to conceive. In July 2017, they had a healthy baby and experienced no complications during the birth [24].

Nonetheless, because pregnancy in the trans male has an important impact on his hormonal balance, it is necessary to find the right time to restart the hormone treatment following the pregnancy. To date, however, there are no studies that explore the optimal time for restarting cross-gender hormone treatment following a pregnancy.

### 3.2. Psychological Aspects

For transgender people who are parents or wish to be parents, being a parent is a key part (or perhaps the most important part) of their identity, and a key role that they experience in their daily life [25]. It is, however, increasingly more common to find trans men who decide to gestate, give birth, and/or breastfeed their babies.

The idea that a trans man can gestate causes a barrier for those health personnel [7] who perceive such an event not possible due to their lack of knowledge [21]. It also supposes an ethical–moral debate because the notion that the act of gestating is exclusive to women still persists socially. During this process, support from family, friends, and a multidisciplinary health team is essential [7,22,26], especially since pregnancy, childbirth, and breastfeeding are processes that can lead the trans person to a state of dysphoria [7,15,21,26,27]. In the case of men who underwent masculinization of the thorax, the dysphoria is less likely to be present due to the decreased development of breast tissue [26]. To decrease the dysphoria, the health team must consider emotional lability as an important factor. Emotional ambivalence is the most characteristic symptom of this state of dysphoria and is related to the hormonal changes. Many of these particular trans men express that they feel like crying all the time [28,29].

Another aspect to consider is the desire for pregnancy. A study conducted among nonhysterectomized trans men, thus trans men who are physically capable of carrying a pregnancy to term, concluded that most of these men were reluctant to become pregnant due to the association of pregnancy with a female identity [30]. At this point, it is important to note that in the studies consulted, the majority of transgender people who have been pregnant parents have had their children before the transition. Among the other options, some consider the pregnancy of their partners, through adoption or embryo preservation, since in many countries hysterectomy is required as a step prior to transition [25].

In many cases, trans men choose adoption. While this could be a personal choice, it is also possible that this important decision is influenced by a lack of information regarding the possibility of preserving fertility [3,9,12,18,20,31]. In a 2016 study conducted by Chen et al., 156 transgender and/or transsexual adolescents with an average age of 16.1 years were surveyed about fertility and the formation of a family. The results revealed that 70.5% of the respondents said they were interested in adoption, while only 35.9% preferred biological parenthood [32]. In a 2017 study, however, Tornello and Bos found that 47% of the surveyed transgender men wanted to have a genetically related child [33]. The discrepancy in the results strongly suggests that adolescents lack information regarding fertility and reproduction and that health services need to consider addressing these issues with adolescents [32]. In a similar study conducted in Australia among 409 individuals, either trans adults or nonbinary people, results similar to those of Chen et al. were obtained, that is, most trans persons prefer adoption over biological parenthood. This finding may be related to the denial of access to fertility services [21]. Accordingly, this study concludes that trans men are having term pregnancies and are doing so without the necessary support and without adequate resources. Such circumstances may cause them to develop feelings of isolation, exclusion, and vulnerability during a critical moment when they desperately need the support of others [21].

If the person has experienced a previous pregnancy that resulted in an abortion, either before or after the transition to FtM, that person may be even more vulnerable to feelings of isolation. A miscarriage is a difficult event to process, and patients often report experiencing confusion and alarm regarding the expectations of a healthy pregnancy, express difficulty in finding meaning to the loss, and have feelings of guilt, emptiness, and a lack of control [34]. These individuals also describe feelings of fear with respect to future pregnancies, are afraid they will not receive the support they need, and believe health providers will not listen to them. In such situations, the need for psychological support that encourages them to express their concerns and fears is extremely important. Similarly, it is important to support and care for the family of the gestating trans individual because they are indispensable agents to the recovery of the patient [35].

The majority of trans people who decide to gestate require some type of assisted reproduction [3,20]. In this respect, it is important to consider the relevance of gestation with his own genetic material or with that of the donor. For some individuals, the inability to conceive children is a stressful event. Even in western society, an important part of adult development and identity is the ability to reproduce. According to the results of Tornello and Boss, where it is shown that 47% of trans people in the study wanted to have children genetically related to them [33], for some individuals, the inability to bear children is perceived as a sign of diminished status and defectiveness [34].

The pregnancy of a trans man forces him to abandon his testosterone treatments [3,7,15,16] and resume a normal menstrual cycle to avoid harming the fetus [3,7,8]. This abandonment of testosterone treatments also causes the person to return, to a greater or lesser extent, to a physical appearance that may be somewhat less masculine. More specifically, because pregnancy in a trans man requires him to be without testosterone for a period of 12 to 16 months, a series of physical changes occurs that impacts one’s sexual identity [21,22,27]. However, after several years of treatment, most of these changes in secondary sexual characteristics are irreversible or only partially reversible. Therefore, during pregnancy, the physical appearance of man is maintained, together with the growth of the uterus, which gives the person the characteristic aspect of pregnancy. The psychological support that the person requires during this period will vary according to the person’s coping strategies at the physical, psychological, and social levels [36].

The experiences of gestational parenthood are diverse. Pregnant transgender men describe various strategies to improve pregnancy coping. Some prefer invisibility, that is, to either go unnoticed as cis-obese men or to impersonate cisgender women. Some want their pregnancy to be known only by their doctors and close relatives to avoid being exposed to increased discrimination and possible transphobic violence [37]. Others, however, prefer to face it normally and be socially visible [37]. Being visible as a transgender man allows them to reaffirm themselves in three areas, specifically, their gender as a man, their identity as a trans, and their status as a pregnant individual. Still for others, it is fundamental for their emotional security and their well-being to be treated as men, including the use of masculine names and pronouns, for example [36].

The actual birthing process is complex, but there is very little information about the delivery process in transgender pregnant men. That said, the data indicate that most deliveries are caesarean [7,15]. This could be due, on the one hand, to the fact that these cases involve high-risk pregnancies in which caesarean delivery is chosen as the best for maintaining control of the situation. On the other hand, trans men may reject vaginal birth, perceiving it as a traumatic and disturbing experience [22]. There are, however, cases where the individual prefers a vaginal birth, a decision that does not presuppose any doubt about their gender identity. Accordingly, it is necessary to inquire about the opinion of each person in this situation, explain the risks and benefits of each of the options, and assess psychologically if the person is prepared for a natural birth, in the event that this is his preference [38].

In 2016, a study conducted by Macdonald et al. described how lactation can produce dysphoria in some pregnant transgender men, while those who wanted to breastfeed described the experience as satisfactory, since they considered it beneficial for the health of their children and for the bonding, attachment, and upbringing with the newborn [26]. Of the 22 participants in the study, nine had undergone chest masculinization. Of those who had not undergone this intervention, 16 attempted to feed their baby, while four of them refused to do so for reasons related to their physical and/or mental health [26]. In any case, deciding to breastfeed or not is a very personal decision [39]. In the event that the person decides to breastfeed, there are multiple support groups that are easily accessible with a large presence on the internet [39,40]. Several testimonies of trans men who have given birth and raised their children without breastfeeding claim that this did not result in sadness or feelings of separation with the baby, but rather, it allowed them to complete an equitable distribution of tasks since, when feeding with baby bottles, the parents can take turns caring for and feeding the baby. This creates a situation of equal importance in parenting and a relationship that challenges the heteronormative roles of the couple [39,41].

### 3.3. Social Aspects

The decision to proceed with a trans pregnancy is framed in a social context that is often complex and hostile, since, in general, transgender people report having experienced numerous cases of discrimination and prejudice [42,43,44]. Some research reveals that more than 75% of trans people have suffered psychological abuses related to gender, and 50% claim to have suffered physical abuse [45,46]. Furthermore, there are studies that indicate that trans people are more likely than homosexual or lesbian people to suffer serious personal injuries, including hospitalization and death [37,47]. The ways in which discrimination against this group manifests are diverse and not always easy to detect.

Such discrimination is evident even in the health field, where trans people are exposed to a high degree of inequality with respect to the care they are provided [48,49]. Specifically, certain countries may place severe restrictions on the reproductive options of trans individuals. Some countries have established sterilization, either through hormonal treatment or surgery, as a requirement to obtain legal recognition of gender. In this context, the act of choosing to have a child as a transgender man is interpreted as an act of transgression, rather than a right [37]. The problem is even more complex because, although research has been developed regarding the gynecological care of trans men, to date, there has been little research on the fertility of and the needs related to pregnancy and maternity/paternity among this population.

In the medical context, the stigma of the trans collective can lead to health professionals offering inadequate information and exhibiting strong microaggressions. This type of behavior, in turn, can cause trans men to avoid seeking medical attention or impede them from revealing relevant information [47]. Invisibility, transphobia, and violence create barriers to the appropriate reproductive care needed by trans people. In addition, transgender people are likely to have specific needs related to fertility, conception, pregnancy, delivery, and the postpartum period that vary substantially from the general population given the biomedical effects from the previous use of exogenous hormones and/or gender affirmation surgeries [37]. That said, it is difficult to make such a critical decision as having a pregnancy when there is a paucity of examples of similar people making the same decision. As a result, it is possible that many trans people are not even able to conceive of this potential life option as a personally viable options from a physical, emotional or social perspective. The lack of visible examples of trans men successfully completing a pregnancy and giving birth can cause health professionals to feel uncomfortable and poorly informed, thereby making it difficult for them to provide adequate care [37]. Thus, education in diversity appears as a central need in the training of health professionals [50,51,52,53,54,55].

In short, the cultural and structural characteristics of our society and institutions, which include anti-transgender stigma, strongly established gender norms regarding pregnancy, institutional structures that do not recognize the possibility of a trans man becoming pregnant, and the lack of research, generate significant difficulties for trans people who want to become pregnant. Unlike the attitudes towards parenthood of gay parents and lesbian mothers, which have been improving over time, there is an extremely low cultural acceptance of trans fathers and mothers and of those whose gender is not clearly defined [2,56].

In a study by Jones (2015) [25], parenthood is an important aspect for trans men who try to solve it by identifying themselves as parents, fathers, uncles, foster parents or otherwise. In this framework, some research indicates that trans parents manifest a high need to demonstrate their competence and adequacy to fulfil a parental role, possibly because they challenge the negative, pathologizing, and devaluating opinions of others and possibly because they are socially excluded from the parental binary father–mother norm [57,58]. In this way, the incursion of trans people into the world of motherhood/fatherhood implies a high degree of readjustment, since their parental roles do not coincide with the conventional roles [37]. For some trans people, it is necessary to conform a normative male role as “husband and father”. This has been used in some theories as a sign of the conservative nature of transgender identity. In this context, the image of a pregnant man presents a significant disruption of the conception of gender as a binary construct. It shows that gender identity is not inherently normative nor inherently dissident [25].

At the same time, there is a need to demonstrate that children of trans parents are not going to have difficulties derived from the family characteristics in which they are raised. In this sense, researchers and academics must start from the cisnormativity and attempt to represent trans families as similar to cisgender families. These studies suggest a defensive position that having a baby in a trans family is normal and that challenging and rethinking the role of gender as a concept is not relevant to the integration and acceptance of trans people in modern western culture. Although this approach may serve to increase the acceptance of trans people as suitable parental figures, it also supports cisnormative expectations for family life and gender socialization between parents and children. This position does not explore whether having a trans parent in a cisnormative society is salient and complex or whether it is negative for the life and family relationships of the children [58].

Another crucial point is the moment to talk with their children and the content they have to communicate. Some trans men had removed oppositional views on whether they should ‘come out’ to their children or the children’s communities when the children were young, with concerns being both for issues of trans visibility and for issues of protecting their children from transphobia which may be levelled against them. There are two clearly different positions. On the one hand, those who are in favor of disclosing do so to reduce prejudice and stigma. They are mostly people of about 20 years, without children, and who identify themselves as transgender. On the other hand, those who are against, are seeking to protect the family and children to avoid discrimination. They are about 30 years old, have children, and identify themselves as men [25].

### 3.4. Legal Aspects

International legislation is extremely diverse with respect to the rights of trans people. In most developed countries, trans people are protected from discrimination by laws that prohibit sex discrimination [59]. Thus, the denial of treatment based solely on gender identity may be prohibited under the laws of discrimination in some states [60]. In several countries, there is no specific prohibition regarding reproduction or parenting by trans persons, although there are no strong policies to protect these rights either. Trans parents face many complex legal issues, including the legal recognition of their name and gender, the validation and recognition with respect to their marriages, the recognition of their legal relationship to their children, and issues regarding child custody [59].

Accordingly, access to assisted reproduction for trans people depends on the legislation of each country, and the countries’ laws regarding trans people differ greatly from each other. There are countries with quite advanced legislation, such as Argentina, which has specific laws and broad rights for transgender people that include access to assisted reproduction [61]. One step behind Argentina are certain European countries such as Malta and Norway. At the other extreme are countries that do not ascribe to the resolutions and declarations of the UN and thus deny individuals the right to freely express their gender identity and sexual orientation [62].

Because of this international diversity, the Principles of Yogyakarta—Principles on the Application of International Human Rights Law in Relation to Sexual Orientation and Gender Identity [63,64,65] is of great interest. The declaration was prepared by a group of human right experts in international law from various countries at the request of the United Nations High Commissioner for Human Rights. The document includes recommendations for governments, government institutions, civil society, and the organization of the United Nations. The Yogyakarta Principles are a universal guide to human rights that affirms binding international legal standards with which States must comply. They are an international legal standard of obligatory compliance for the States, before which some countries have expressed their reservations [66].

Some of these principles are of special application in the case of access to assisted reproduction techniques for trans people. Principle 13, The right to social security and other social protection measures affirms that “everyone has the right to social security and other social protection measures, without discrimination on the basis of sexual orientation or gender identity”. Principle 17, The Right to the Highest Attainable Standard of Health, affirms that “everyone has the right to the highest attainable standard of physical and mental health, without discrimination based on sexual orientation or gender identity. Sexual and reproductive health is a fundamental aspect of this right”. Related to this principle, the Yogyakarta plus10 affirms that “States that enforce that legal provisions, regulations or any other administrative measures on the donation of blood, gametes, embryos, organs, cells or other tissues do not discriminate on grounds of sexual orientation, gender identity, gender expression or sex characteristics”. Principle 24, The Right to a Family, affirms that “everyone has the right to find a family, regardless of sexual orientation or gender identity. Families exist in diverse forms. No family may be subjected to discrimination on the basis of the sexual orientation or gender identity of any of its members. According to that principle, States shall take all necessary legislative, administrative and other measures to ensure the right to a family, including through access to adoption or assisted procreation (including donor insemination), without discrimination based on sexual orientation or gender identity”. Among the Yogyakarta plus10, Principle 24 affirms that States will “enable access to methods to preserve fertility, such as the preservation of gametes and tissues for any person, without discrimination on grounds of sexual orientation, gender identity, gender expression, or sex characteristics, including before hormonal treatment or surgeries, and ensure that surrogacy, where legal, is provided without discrimination based on sexual orientation, gender identity, gender expression or sex characteristics” [64,65]. These principles intend to recommend and compel States to direct legislation, jurisprudence, and doctrines on the subject. The intent of these principles is the systematic and detailed reflection within the sphere of international law [63].

The legislative and regulatory framework, apart from being heterogeneous, is complex. It is not easy for the researcher in health sciences to find evidence of the legal state of the world situation regarding assisted reproduction in trans people. Thus, providers should encourage trans patients to consult appropriate professionals to become informed about the legal issues involved in becoming a parent through assisted reproduction techniques in their own countries [59].

In Spain, the country where Dan’s story takes place, the legislation is quite advanced within the European environment [67]. According to Spanish legislation, the legal right to change names and gender is recognized providing the requirements of being of legal age and having the diagnosis of gender dysphoria are met. In addition, the trans person must have at least two years of hormonal treatment or have undergone a sexual reassignment surgery [68]. Within this Spanish legal context, the issue of assisted reproduction arises as a fertility treatment for couples in which at least one of the members is trans. Law 14/2006 (May 26) addresses techniques of assisted human reproduction and provides in its third article that “assisted reproduction techniques will be applied only when there are possibilities of success that do not suppose serious risk to the health, physical or mental, of women”. The law also establishes a National Commission for Assisted Human Reproduction (CNRHA) as an organ of the Ministry of Health, Social Services and Equality that is responsible for establishing homogeneous and equal criteria regarding the access of trans people to these treatments [1]. From this law, it can be deduced that neither orientation nor sexual identity can be used as conditioning factors to deny the aforementioned groups access to assisted reproduction treatments. This same law, in its sixth article, proclaims that “all women older than 18 years and with full capacity to act, may be a recipient or user of the techniques provided for in the Act, regardless of their marital status and sexual orientation”.

Nonetheless, as already mentioned, this legislation is not common to all countries. Even with advanced legislation, such as in Spain, there are aspects to which the law does not give a response, thus leaving their resolution to the ethical considerations of experts and committees whose findings are not always fair. For example, one such finding is that a trans person, after his transition, is not legally considered a woman even with his female reproductive organs. This is recorded in his civil registry after having undergone hormonal treatment. The same law that protects him by recognizing his sexual identity discriminates against him by not allowing him to be a potential beneficiary of assisted reproduction techniques. According to this, the person must be a woman to be entitled to these techniques, in contravention of gender discrimination.

Another aspect involves the financing of procedures. According to Royal Decree-Law 1030/2006 (September 15), which establishes the portfolio of common services of the National Health System, assisted human reproduction treatments will not be financed to people who have undergone a voluntary sterilization treatment [69]. That is, trans men who, by personal or medical decision, have undergone an oophorectomy, cannot benefit from an assisted reproduction treatment financed by the public system. Again, this population is discriminated against, leading to costly fertilization treatments that they must pay in the event they want to have offspring of their own.

Finally, consideration should be given to the role of the health care ethics committees as they are not part of the legal terrain, but rather, they condition decisions about whether a trans person can or cannot access paternity. The scientific debate on fetal viability due to the teratogenic effects of testosterone must be considered and is one of the most important issues to these committees. Despite the existence of scientific publications and a clear record of healthy children born to trans men after abandoning hormone treatment [59], there is still doubt about the viability of these embryos, whether they are their own or are from donors. This means that the local ethical committees of the hospitals are the ones who decide on the possibility of a trans man carrying out a pregnancy according to Law 14/2006, where it is stated that the techniques of assisted reproduction will be accessible only when there are reasonable possibilities of success [1].

In a study conducted by the American Society for Reproductive Medicine, the ethics committee concluded that trans identity/status by itself should not necessarily include fertility preservation and assisted reproductive services. Professional autonomy, while a significant factor when deciding who to treat, is limited in this case by a greater ethical obligation to regard persons, regardless of their gender identity [59]. The same conclusion could be made in any other country, including Spain, and applied to Dan’s case.

## 4. Discussion

The results of this review showed that trans parenthood has impact in four areas: Biological, social, psychological, and legal. In general, the available evaluations of transparenthood reveal that research on transgender paternity is scarce [2,4], although there are more and more technical resources and more sources of information on how to be parents [25,26,30,31,36,37,39,45]. However, many FtM people were parents and expressed frustration at the overlooked of their ability to do so, which is an important element for their identity [25].

There are no biological, psychological, social or legal constraints or rules preventing a trans person from gestating, giving birth, and breastfeeding their offspring. There is nothing preventing it, but there are challenges. Hence, it is the responsibility of health professionals to fully support the trans individuals who choose gestation and provide them with all the necessary information they will need as they move forward to achieve their goal [3,7,15,20,21]. Male gestation involves gestation by the trans man with his female reproductive organs. However, to accomplish this, hormone treatment must be temporarily suspended so that the menstrual cycle reappears [7,8]. The problem with this type of pregnancy is that it is quite unusual, and thus, there are few documented cases and no long-term follow-up studies of the trans individual or the child [7,15,20,21,25].

The possible biological problems derived from the use of testosterone are fewer than the problems derived from the weight of society and the individual’s own feelings to accept the dysphoria that the trans person may face [28,29,36,37]. The gestation of a trans male is a biological, psychological, and social challenge. This fact supposes a radical rupture when associating parenthood with biology and nature [36,37].

The medical and technological advances regarding the different reproductive options favor the trans collective to go to centers specializing in assisted reproduction techniques; doing so is also impacted because this group is often invisible in public. The lack of visibility of the reproductive diversity of trans people means that many trans people do not even consider the reproductive options available to them [64,65,66]. Moreover, this lack of visibility means that health professionals do not have clear references or access to the trans population and are therefore hindered in their attempts to intervene, care for, and assist, from a comprehensive perspective, trans people seeking medical information and help [15,20,22,26,31]. In addition, the legal void in which transgender people find themselves with respect to their reproductive possibilities produces an additional difficulty in accessing these techniques [59,60,61,62,66].

Currently, although gestation is legal in men and there is increasing effort to raise awareness among the population so that it is perceived as something completely normal, the concept is far from being fully accepted. A part of society that still does not agree or accept the practice is often motivated by transphobia, which can lead to discriminatory practices that affect the advancement of trans person gestation [47,48,49]. Although these cases have revolutionized the panorama of family life and daily life, they often give rise to tension among people with more conservative thoughts [21,24,25,26].

In this literature review, the authors present an integral perspective of trans person gestation whereby trans people who are facing assisted reproduction techniques must struggle with all these facts, with one influencing the other in a holistic life experience of parenthood. Dan’s case is an example of how many factors can influence this difficult decision.

The results of this study suggest that there are not always biological and legal barriers preventing a trans man from having biological offspring. There are examples of trans men who have completed pregnancies and produced completely healthy children. In the words of Thomas Beatie when speaking to Oprah Winfrey, “I have a very stable male gender identity. I see pregnancy as a process, and it does not define who I am. It is not a male or female desire to want to have a child—it is a human desire. I’m a person, and I have the right to have my own biological child” [24].

Three limitations emerge from this study. The first is that it is not a systematic review; it is a revision of the literature based on the problematic of a specific case. Therefore, there may be documents that have not been incorporated. Nor has a measure been made of the quality of the articles found or the degree of evidence they generate. More depth of research is needed in this area. The wide range of factors and their variability among different cultures and countries (especially in legal and cultural factors as well as access to healthcare) must also be considered, making it difficult to synthesize concrete and extrapolatable ideas to larger populations. Another limitation is that in the studies consulted, most people have not had children before or have had them before the transition. Despite these limitations, literature-based arguments have been gathered for the transparenthood in the biological, social, psychological, and legal context.

As future directions for research, the results of this review identified several biological, social, psychological, and legal factors that impact a trans person’s ability to parenthood. Among them, some are negative, so research to find strategies for how to mitigate these factors needs to be explored. Paternity assumes new roles and areas of responsibility specifically defined, and more research is needed to consider trans parenthood holistically.

## 5. Conclusions

There are different forms of trans parenthood, including biological gestation by the FtM person. The biological paternity, once the person makes the transition (central idea of this study), is unusual, and there are few documented cases and no long-term follow-up studies of the trans individual or the child.

The literature highlights many of the factors that continue to serve as facilitators or barriers for transparenthood. There is no biological, psychological, social or legal constraints or rules preventing trans from gestating, giving birth and breastfeeding their offspring.

International legislation is extremely diverse with respect to the rights of trans people, especially about paternity in general and gestation in particular.

The lack of visibility of the reproductive diversity of trans people means that many trans people do not even consider the reproductive options available to them.

A part of society that still does not agree or accept the practice is often motivated by transphobia. It is a disruption to the very heart of the binary gender construct.

Deciding how, when, and the disclosure (or not) of one’s trans status to one’s children is considered a challenge, with significant pros and cons in both sides. These positions are related to other variables, such as age and parenthood.

This review also highlights the fact that these factors are often interrelated and somewhat fluid in categorization, with several factors overlapping in multiple categories. The recommendations that arise from this review provide argument and support to show that trans people can perform effective parenthood, with a good control of all these factors.

There is a growing need for more attention and research in this area.
